# Growing of Artificial Lignin on Cellulose Ferulate Thin Films

**DOI:** 10.1021/acs.biomac.2c00096

**Published:** 2022-04-19

**Authors:** Thomas Elschner, Jörg Adam, Hans Lesny, Yvonne Joseph, Steffen Fischer

**Affiliations:** † Institute of Plant and Wood Chemistry, Technische Universität Dresden, Pienner Str. 19, Tharandt 01737, Germany; ‡ Institute of Electronic and Sensor Materials, TU Bergakademie Freiberg, Gustav-Zeuner-Str. 3, Freiberg 09599, Germany

## Abstract

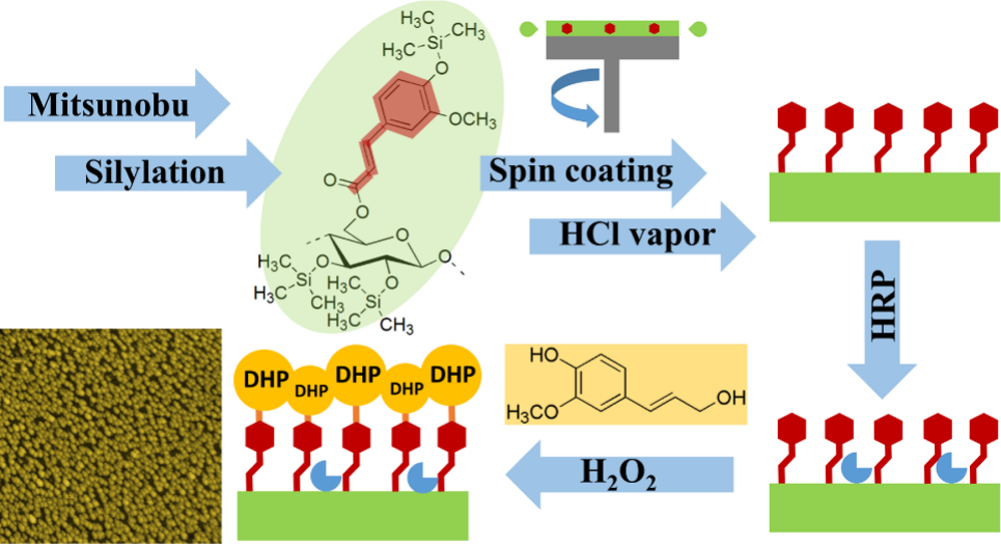

Thin films of cellulose ferulate were designed to study the formation of dehydrogenation polymers (DHPs) on anchor groups of the surface. Trimethylsilyl (TMS) cellulose ferulate with degree of substitution values of 0.35 (ferulate) and 2.53 (TMS) was synthesized by sophisticated polysaccharide chemistry applying the Mitsunobu reaction. The biopolymer derivative was spin-coated into thin films to yield ferulate moieties on a smooth cellulose surface. Dehydrogenative polymerization of coniferyl alcohol was performed in a Quartz crystal microbalance with a dissipation monitoring device in the presence of H_2_O_2_ and adsorbed horseradish peroxidase. The amount of DHP formed on the surface was found to be independent of the base layer thickness from 14 to 75 nm. Pyrolysis-GC-MS measurements of the DHP revealed β-O-4 and β-5 linkages. Mimicking lignification of plant cell walls on highly defined model films enables reproducible investigations of structure–property relationships.

## Introduction

Utilization of lignocellulosic biomass, for example, from wood, grasses, or agricultural residues, is the future basis for renewable materials and energy supply. In the cell wall of woody plants, cellulose, hemicellulose, and lignin are the main components combined together in sophisticated hierarchical structures.^1^ However, the separation of cell wall components by pulping including pretreatments, enzymatic hydrolysis,^2^ and general biorefinery^3^ is of huge interest, and several approaches in basic and applied research are popular. Investigations on natural plant cell walls are very difficult due to their complex architecture and, thus, model compounds are promising in order to study structure–property relationships.

Nanometric thin films based on cellulose are predestinated for studies on well-defined surfaces since they are suited for adsorption studies^4^ and could also be tailored by chemical post-modification.^5,6^ Ultrathin films of cellulose were applied as model systems and in field of advanced materials.^7^ Very smooth films are obtained by spin coating or the Langmuir–Blodgett technique of trimethylsilyl (TMS) cellulose and subsequent regeneration with hydrochloric acid vapor.^8,9^ It is up to researchers to create more complex plant cell wall models based on cellulose thin films, for example, by inclusion of lignification processes.

Mimicking the lignification in plant cell walls by dehydrogenative polymerization of monolignols was carried out in vitro by Freudenberg^10^ for the first time. Dehydrogenation polymers (DHPs, i.e., synthetic lignins) are typically obtained by oxidative coupling of hydroxycinnamyl alcohols (sinapyl-, coniferyl-, and coumaryl alcohol) applying a peroxidase with hydrogen peroxide. In subsequent studies, reaction conditions were tuned including effects of polysaccharide matrices. For instance, pectin improves the dispersion of DHP in the cellulose network, enhancing the formation of aryl–aryl ethers.^11^ Cyclodextrin increases the β-O-4-content, whereas β-5-linkages are decreased.^12^ Moreover, the influence of hemicellulose on molecular and supramolecular structures of DHPs synthesized in bulk was investigated.^13^ However, lignification experiments at interfaces usually do not allow a distinction between adsorption of DHP on the polysaccharide surface and the formation of covalent bonds.

Until now, the formation of synthetic lignin on planar surfaces is mostly limited to adsorption studies, for example, deposition of DHP on hemicellulose films.^14,15^ Wang et al. performed real-time experiments on QCM-D, that is, dehydrogenative polymerization of hydroxycinnamyl alcohols was carried out on gold and silica surfaces and films of cellulose nanocrystals.^16^ However, it would be advancing to use model surfaces with anchor groups, such as ferulate moieties, present in plant cell walls, which could be artificially lignified.^17^

In the present work, approaches from online monitoring by QCM-D and polymerization of monolignols from a surface decorated with ferulate moieties were combined. TMS cellulose ferulate was synthesized by advanced synthetic chemistry utilizing the Mitsunobu reaction. The solubility of this biopolymer derivative in easily evaporable solvents allows tailoring of nanometric thin films by spin coating. Those synthetic model films enable defined artificial lignification to study structure–property relationships.

## Experimental Section

### Materials

Microcrystalline cellulose (Avicel PH-101) possessing a  of 330 and a water content of 3.2% was purchased from Sigma-Aldrich and dried at 60 °C under vacuum. Lithium chloride (Carl Roth) was dried at 100 °C under vacuum before use. Coniferyl alcohol (98%) was received from Alfa Aesar. ABTS [diammonium salt of 2,2′-azino-bis(3-ethylbenzothiazoline-6-sulfonic acid)] from Roche Diagnostics (Mannheim, Germany) was used. Horseradish peroxidase (HRP) from Merck (Darmstadt, Germany) possessed an activity of 251 U mg^–1^ (ABTS assay). TMS cellulose (DS_NMR_ 2.85) was synthesized, according to a procedure by Kostag et al.^18^ applying *N*,*N*-dimethylacetamide (DMA)/LiCl as the solvent. Other chemicals were purchased from Sigma-Aldrich, VWR, or Carl Roth and were used without further treatment.

Silicon wafers (P/Bor, 100 mm diameter, 525 μm thickness, <100> orientation) were purchased from Si-Mat (Kaufering, Germany) and cut into 15 mm × 15 mm pieces. Quartz crystal microbalance with dissipation monitoring (QCM-D) sensors with a gold layer (QSX301) were purchased from Quantum Design (Darmstadt, Germany). Ultrapure water possessing a resistivity of 0.05 μS cm^–1^ was used for surface experiments.

### Measurements

NMR spectra were recorded on a Bruker Avance Neo 700 MHz SB instrument (up to 80 mg mL^–1^ sample, 10 000 scans) in CDCl_3_ or dimethyl sulfoxide (DMSO-d_6_). Relaxation delay was set to 10 s for quantitative evaluation of the ^1^H NMR experiments. Fourier transform infrared (FTIR) spectra were recorded on a FTIR spectrometer Tensor27 (Bruker Optics GmbH).

Water contact angles (CAs) were determined with a Dataphysics instrument (Filderstadt, Germany) with the sessile drop method and a drop volume of 3 μL. The photographic images were evaluated with Python software using the pyDSA package for drop shape analysis. Measurements were performed at room temperature and were repeated at least 10 times.

Atomic force microscopy (AFM) was performed in the contact mode with a TMX 2010 instrument (TopoMetrix, now Bruker). The images were scanned using cantilevers CSC12/Si_3_N_4_ (MikroMasch, Estonia) with a resonance frequency of 10 kHz and a force constant of 0.03 Nm^–1^. Images were processed using Gwyddion software applying plane leveling, alignment of rows, correction of horizontal error lines, and fix zero. RMS (Sq) values were obtained from statistical sizes of processed AFM images (10 μm × 10 μm).

QCM-D experiments were carried out with a Q-Sence E1 instrument (Gothenburg, Sweden) at 23.0 °C, applying a flow rate of 100 μL min^–1^. The relative resonant frequency (Δ*F*) and the relative dissipation factor (Δ*D*) of the third overtone (*n* = 3) were determined in comparison to the zero values. The fundamental frequency of quartz crystals is *f*_0_ ≈ 5 MHz, and the sensitivity constant *C* = 17.7 ng Hz^–1^ cm^–2^.

To determine the layer thickness of thin films, QCM-D sensors were measured in air in uncoated, spin-coated, and desilylated state. The measurements were performed until a stable baseline was obtained. A baseline is considered to be stable if Δ*F* is changing less than 1 Hz in 10 min. The data files of individual measurements were stitched together to obtain the change in the frequency. Due to the rigidity of evenly distributed films, the Sauerbrey equation is valid, describing a linear relationship between frequency change and adsorbed mass.^19^ Areal mass density of the adsorbed film (*m*_film_) was calculated from this Sauerbrey relationship using the following equation^20^
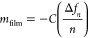
1

The overtone number (*n*) is automatically considered by software. Film thicknesses (*d*) were calculated from *m*_film_ and film densities (ρ_film_) of cellulose and TMS cellulose films, according to the literature.^21^

Flow through QCM-D experiments were started by rinsing of the sensors with ultrapure water for about 1 h to obtain a stable baseline when restarting the measurement. After 10 min, HRP (1 mg mL^–1^) was introduced into the flow cell for 10 min. Subsequently, the excess of loosely bound enzyme was removed by rinsing with H_2_O for 20 min. The polymerization was performed with an aqueous solution composed of 20 mM H_2_O_2_ and 0.5 mg mL^–1^ coniferyl alcohol for 40 min. Afterward, the sensors were rinsed for 30 min with H_2_O. Δ*F* was evaluated at 40 and 110 min of the experiments to exclude bulk effects from H_2_O_2_ and coniferyl alcohol. The Sauerbrey equation is not valid for viscoelastic layers that were formed by polymerization of coniferyl alcohol. Monitoring Δ*F* and Δ*D* provides information about rigidity or softness of the films. The dissipation factor (*D*) is the ratio between dissipated and stored energy in the oscillating system^22,23^
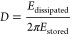
2

As a rule of thumb, films can be considered as rigid if the ratio of Δ*F* and Δ*D* is >25.^24^

Py-GC-MS was performed on an Agilent system (GC 7890 B/MSD 5977). Samples (about 300 μg) were yielded from silicon wafers by scraping off the film with a razor blade and pyrolyzed by a Multi-Shot Pyrolyzer EGA/PY-3030D (Frontier Lab) at 450 °C. Separation was achieved by a ZB-5MS capillary column (30 m × 0.25 mm) with a temperature program of 50–240 °C at 4 K min^–1^. NIST MS Search 2.2 (2014) software was used to identify compounds by comparing spectra in the NIST MS library.

### Synthesis of Cellulose Ferulate

Cellulose (3 g, 18.5 mmol) was stirred in dry DMA (90 mL) for 2 h at 120 °C. After cooling the suspension to 90 °C, LiCl (5.4 g) was added, and a clear solution was obtained by continued stirring. Ferulic acid (3.60 g, 18.5 mmol) was added and dissolved completely in the solution of cellulose. Afterward, triphenylphosphine (4.86 g, 18.5 mmol) was added, and the resulting solution was cooled to 0 °C by an ice bath. Subsequently, diisopropyl azodicarboxylate (DIAD) (3.75 g, 18.5 mmol) was added dropwise under stirring. Furthermore, the reaction mixture was stirred at room temperature for 2 d. To yield the crude product, the material was precipitated into methanol (1200 mL) and was washed three times with methanol (600 mL), two times with water (600 mL), and finally with methanol (600 mL). The raw product was dried under vacuum at 40 °C. For analytical purposes, the material was reprecipitated from DMSO in methanol.

Cellulose ferulate (**2**), degree of substitution (DS) 0.35; ^13^C NMR (700 MHz, DMSO-d_6_): δ [ppm] = 164.3 (C=O), 151.4, 149.9, 149.3, 148.0, 147.4, 146.3, 145.3, 141.4, 132.8, 125.5, 123.5, 122.2, 117.1, 115.6, 114.1, 113.0, 112.3, 111.4, 102.9 (C1), 80.3 (C4), 74.8 (C5/C3), 73.0 (C2), 63.3 (C6s), 60.2 (C6), 56.1, 55.2 (OCH_3_).

### Synthesis of TMS Cellulose Ferulate

Cellulose ferulate (3.0 g, 13.4 mmol) was suspended in dry DMA (50 mL), and hexamethyldisilazane (HMDS) (31 mL, 150 mmol) and trimethylsilyl chloride (TMSCl) (200 μL, 1.6 mmol) were added. The reaction mixture was stirred for 1 h at 80 °C, cooled to room temperature, and precipitated into deionized water (1.2 L). The raw product was washed with water until the yellow color disappeared. Subsequently, the product was dried under vacuum at 40 °C to yield about 5 g of solid. For further purification, TMS cellulose ferulate was dissolved in ethyl acetate (70 mL), and insoluble impurities were removed by centrifugation. The clear solution was poured into aqueous sodium hydrogen carbonate (5 g in 1.2 L water), and the product was filtered off, washed with water (four times 400 mL), and finally dried under vacuum at 40 °C.

TMS cellulose ferulate (**3**), yield: 3.66 g (9.01 mmol, 67%), DS_ferulate_ 0.35, DS_TMS_ 2.53; ^1^H NMR (700 MHz, CDCl_3_): δ [ppm] = 5.6–8.2 (CH_ferulate_), 2.5–5.0 (CH_AGU_, OH_AGU_, OCH_3_), −1.0–1.0 (TMS).

### Determination of DS Values by ^1^H NMR Spectroscopy

Cellulose ferulate (300 mg) was suspended in pyridine (5 mL), and acetic anhydride (5 mL) was added. The reaction mixture was stirred for 24 h at 60 °C. Afterward, insoluble impurities were centrifuged off. The product was yielded by precipitation into deionized water (150 mL) containing NaHCO_3_ (0.5 g). Purification of the materials was achieved by filtration, washing three times with deionized water (100 mL), and drying under vacuum at 40 °C.

Peracetylated cellulose ferulate, FTIR: no ν_OH_, ^13^C NMR (700 MHz, CDCl_3_): δ [ppm] = 170.2 (6_C=O_, Ac), 170.0 (C=O, ArOAc), 169.7 (2_C=O_, Ac), 169.3 (3_C=O_, Ac), 164.5 (C=O), 151.7 (C–OMe), 146.3 (CH-9), 141.9 (C-OAc), 133.0 (C10), 123.5, 121.6, 116.8 (CH-8/15/16), 111.5 (CH-11), 100.5 (C1′), 76.1 (C4), 72.8 (C5), 72.5 (C3), 71.8 (C2), 62.0 (C6s), 56.1 (OCH_3_), 20.8 (CH_3_ Ac), 20.7 (CH_3_ ArOAc), 20.6 (CH_3_ Ac), 20.5 (CH_3_ Ac).

The DS_ferulate_ value was calculated from integral intensities (*I*) of ^1^H NMR signals of CH-protons (ferulate, low field, 8.0–6.0 ppm) and CH_3_-protons (acetate, high field, 2.5–1.0 ppm) with the following equation
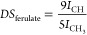
3

The DS_TMS_ value was calculated from integral intensities (*I*) of ^1^H NMR signals of CH_3_-protons (TMS, high field, −1.0–1.0 ppm) and CH-protons (ferulate, low field, 5.6–8.2 ppm) including DS_ferulate_ with the following equation
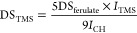
4

### Preparation of Films

Silicon wafer pieces were pre-cleaned with 2-propanol for 10 min in an ultrasonic bath, rinsed with water, and treated with piranha solution [15 mL of H_2_SO_4_ (96%) and 5 mL of H_2_O_2_ (35%)] for 1 h. Subsequently, surfaces were rinsed with ultrapure water and dried with nitrogen gas. QCM-D crystals were cleaned with Radio Corporation of America (RCA) solution [10 mL of H_2_O, 2 mL of NH_3_ (25%), and 2 mL of H_2_O_2_ (35%)] at 75 °C for 15 min and finally rinsed. The support materials were spin-coated at 4000 rpm (acceleration 2500 rpm/s) for 60 s using a POLOS SPIN150i-NPP single substrate spin processor (Desktop Version) from SPS-Europe B.V. (Putten, Netherlands). For cleavage of silyl groups, films were treated with hydrochloric acid vapor (from 10 wt % HCl) in Petri dishes for 15 min at room temperature. This procedure was adapted from the literature describing desilylation of TMS cellulose films.^8^

### Determination of Enzyme Activity

Activity of HRP in solution and on surface was determined by ABTS assay. The oxidation product [ABTS*]^+^ was quantified by a UV–vis spectrometer V-650 (Jasko) at 405 nm. To measure the appropriate absorption values and to perform the convenient reaction kinetics, ABTS (5 mM), H_2_O_2_ (5 mM), and HRP (5 × 10^–6^ mg mL^–1^) in ultrapure water were applied. For determination of HRP activity on the surface, a piece of wafer (**Film 2** with HRP, 2.25 cm^2^) was shaken in a solution of reagents (20 mL). HRP activity was calculated from the slopes of the time-dependent absorption utilizing the Beer–Lambert law. The extinction coefficient ε of [ABTS*]^+^ is 27.5 L mmol^–1^ cm^–1^ and was taken from the literature.^25^

## Results and Discussion

Recently, we found a novel synthesis path for cellulose esters of hydroxycinnamic acids under Mitsunobu conditions.^26^ The reaction conditions were successfully transferred from advanced organic chemistry to polymer-analogous modifications. This highly selective esterification is tolerant regarding double bonds and phenolic hydroxyl groups. Thus, utilization of protecting groups is not required. Moreover, drawbacks of Steglich esterification, that is hardly removable dicyclohexylurea and poor solubility of products, could be avoided.

### Synthesis of TMS Cellulose Ferulate

To prepare the cellulosic thin films possessing ferulate moieties by spin coating, soluble derivatives are required. Therefore, the synthesis of DMSO-soluble cellulose ferulate under Mitsunobu conditions was applied.^26^ However, the low vapor pressure of DMSO limits the formation of nanometric films. In this regard, cellulose ferulate was silylated with HMDS to enable processing in easily evaporable organic solvents.

**Figure 1:**
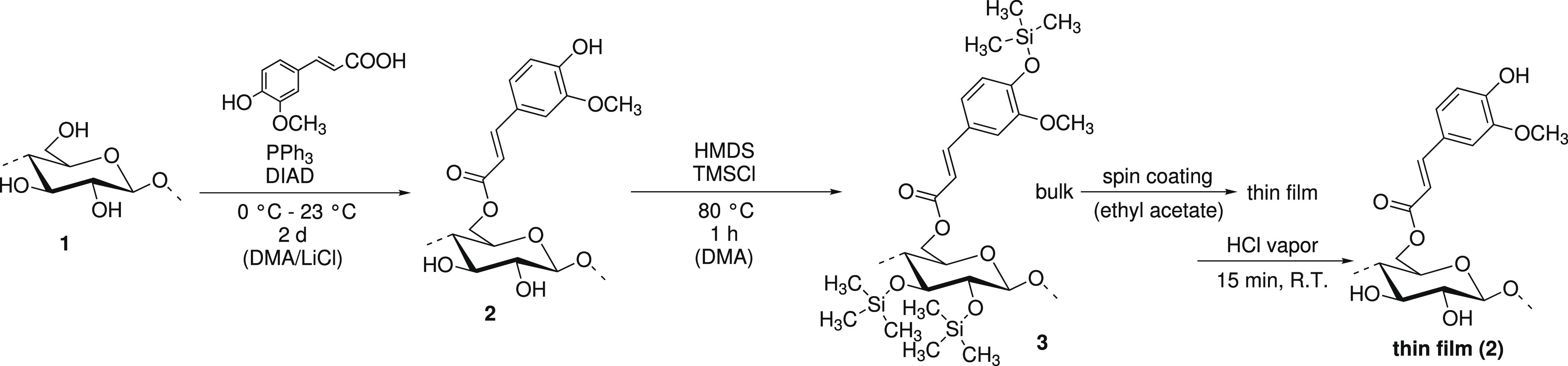
Reaction scheme for synthesis of TMS cellulose ferulate, subsequent film formation, and desilylation.

In the first step, cellulose (**1**, Figure 1) dissolved in DMA/LiCl was converted with ferulic acid, triphenylphosphine, and DIAD at 0 °C. The mixture was allowed to warm up to room temperature and stirred for 2 d to yield cellulose ferulate (**2**) with a DS value of 0.35. In a further step, cellulose ferulate was converted with an excess of HMDS in the presence of TMSCl as the catalyst using DMA as the solvent. This procedure was adapted from trimethylsilylation of pure cellulose.^18^ The TMS cellulose ferulate (**3**) possessed DS values of 0.35 (ferulate) and 2.53 (TMS). It was soluble in ethyl acetate and chloroform but insoluble in acetone.

### Molecular Structure Characterization of Bulk Materials and Determination of DS Values

Molecular structure of cellulose ferulate (**2**) could be clearly revealed by FTIR and NMR spectroscopy and was published in detail previously.^26^ In the IR spectrum (Figure S1, top), aliphatic −C–H vibrations are indicated at 2884 cm^–1^. The −C=O signal of the ester moiety appears at 1722 cm^–1^. Signals at 1632, 1594, and 1510 cm^–1^ reveal aromatic structures. The ^13^C NMR spectrum clearly shows the linkage of ferulate to cellulose backbone by the carbonyl signal at 164.3 ppm (Figure S2). The resonances of CH moieties from aromatic rings and double bonds are detected between 111 and 152 ppm. Peaks of the modified anhydroglucose unit (C1–C6) are in typical range of cellulose esters. The methoxy group is indicated at 56 ppm.

**Figure 2:**
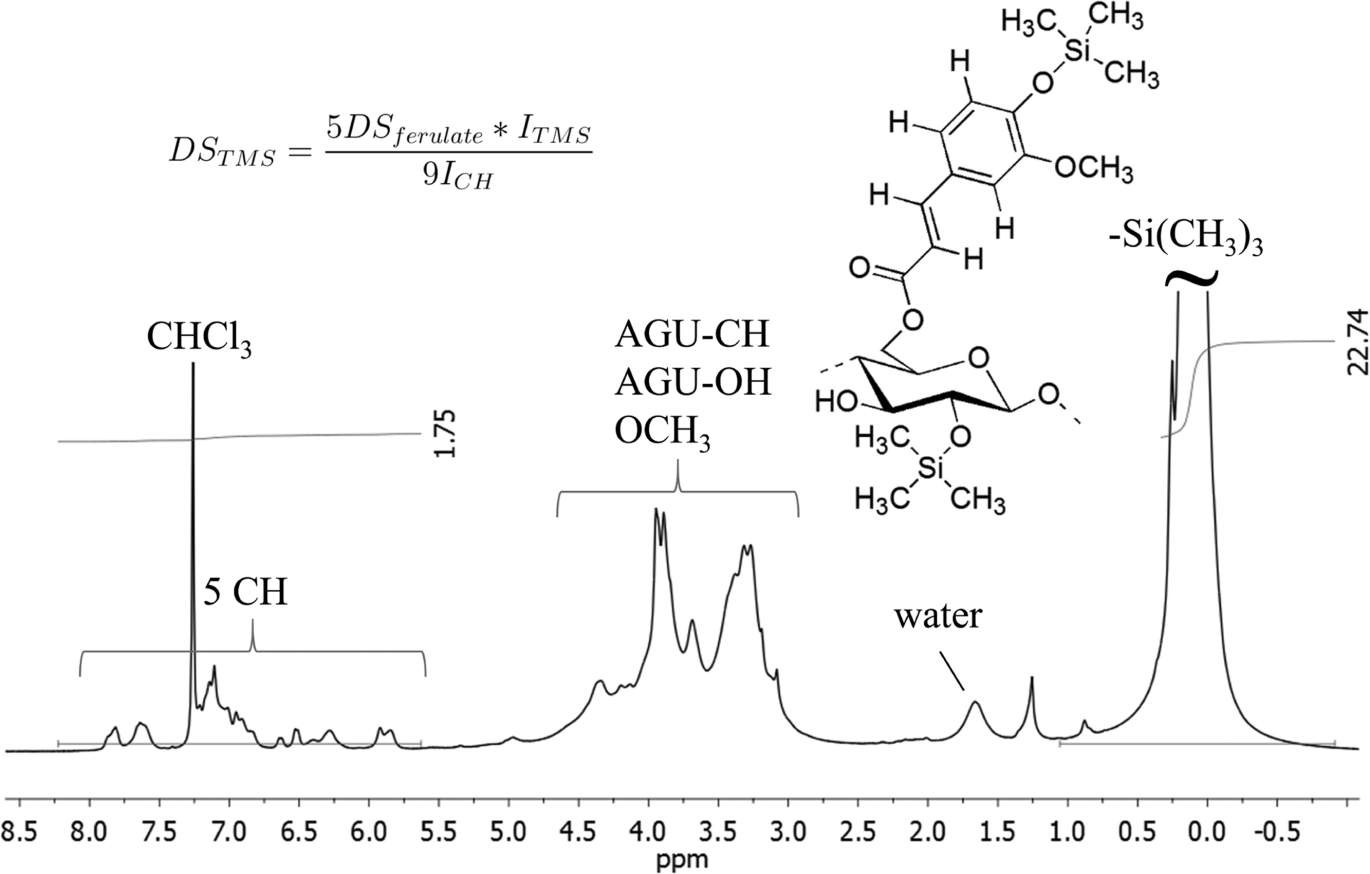
^1^H NMR spectrum of TMS cellulose ferulate (**3**) with illustration for determination of the DS_TMS_ value.

The IR spectrum of trimethylsilylated cellulose ferulate (**3**, Figure S1, bottom) shows a strongly decreased OH stretching but increased aliphatic -C-H vibrations arising from substitution with TMS groups. Moreover, a typical peak of the Si–CH_3_ vibration at 1250 cm^–1^ occurs. The ^1^H NMR spectrum shows CH moieties from aromatic rings and double bonds at 5.6 to 8.2 ppm (Figure 2). Resonances of CH and OH groups arising from the modified anhydroglucose unit and methoxy group are overlapping from 2.5 to 5.0 ppm. The maximum of a very strong TMS signal is at 0 ppm. ^13^C NMR spectrum of **3** does not provide additional information since the resolution is poor. However, the key resonances of substituents are visible.

The DS_ferulate_ value of cellulose ferulate (**2**) was calculated from the integral intensities of the ^1^H NMR spectrum of the peracetate.^26^ Calculations are based on the ratio of CH moieties appearing in the low field from 8.0 to 6.0 ppm and methyl groups of acetate in the high field from 2.5 to 1.0 ppm. A complete acetylation of hydroxyl groups can be assumed due to missing OH valence in the IR spectrum.

The DS_TMS_ value of TMS cellulose ferulate (**3**) was calculated from the ratio of intensities of CH moieties (low field) and the TMS signal (Figure 2). Moreover, the DS_ferulate_ value was used, as determined from the peracetate.

### Thin-Film Formation

To obtain nanometric films possessing ferulate anchor groups for artificial lignin polymerization, TMS cellulose ferulate (**3**) was spin-coated followed by removal of silyl groups with HCl vapor (Figure 1). In the first set of experiments, **3** was dissolved in ethyl acetate and spin-coated at defined rotation settings but varying polymer concentrations. It turned out that smooth and uniform films can be obtained from solutions with a mass concentration (β) of 5 to 25 mg mL^–1^. The film obtained with β = 10 mg mL^–1^ was used in extended experiments and is termed **Film 3**, and its desilylated material is **Film 2**.

**Figure 3:**
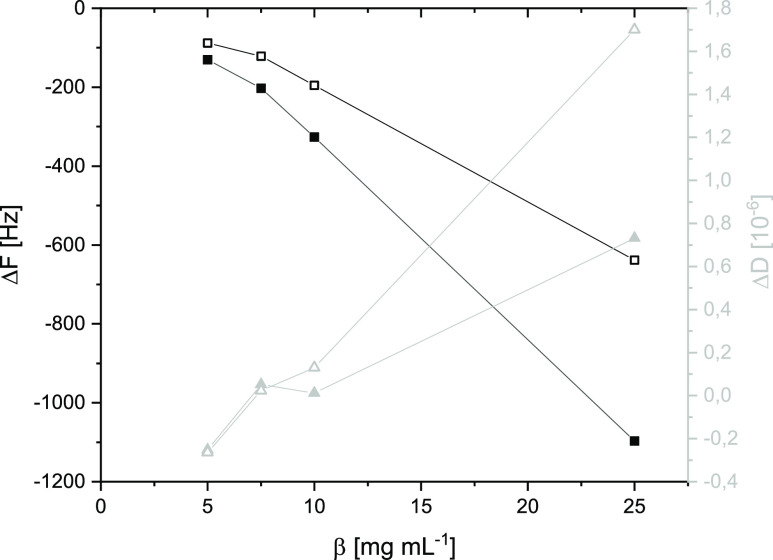
Equilibrium values of QCM-D experiments of dry films in air: Δ*F* (black squares) and Δ*D* (gray triangles) depending on the mass concentration used for spin coating. Films of TMS cellulose ferulate are indicated with filled symbols. Films of cellulose ferulate obtained by desilylation are marked with hollow symbols.

Film thicknesses of TMS cellulose ferulate films and cellulose ferulate films were determined by QCM-D measurements in air using equilibrated values. The same sensors were measured before and after coating, as well as after desilylation. Individual baselines of the measurements were stitched together to obtain frequency shifts (Δ*F*) and the change in the dissipation factor (Δ*D*). In general, dissipation is expressed as the ratio of dissipated and stored energy when the film on the sensor is subjected to the oscillating cycle (eq 2 of the experimental section). Cellulosic thin films behave fully elastic, that is, deformations are fully reversible, indicated by approximately no change in dissipation. In this case, there is a linear relationship between Δ*F* and adsorbed mass, according to the Sauerbrey equation^19^ (eq 1 of the experimental section). Figure 3 shows Δ*F* and Δ*D* values depending on the polymer concentration used for spin coating. As expected, Δ*F* decreases, and thus areal mass density of the films (*m*_film_) increases with increasing concentration. Thicknesses were calculated from *m*_film_ and thin film density (ρ_film_) from the literature. Assuming a film density of 1.5 g cm^–3^ for cellulose films,^21^ layer thickness (*d*) is in the range of 10 nm to 75 nm. A typical example for the calculation of areal mass density and thickness from frequency shifts Δ*F* can be considered in Table 1.

**Table 1:** Analysis of Thin Films: Δ*F* Values Obtained from QCM-D Measurements Generate Film Thicknesses (*d*) Calculated from Areal Mass Density of Film (m_film_) and Film Density (ρ_Film_, Literature^21^)^a^

sample no	Δ*F* [Hz]	*m*_film_ [μg cm^–2^]	ρ_film_ [g cm^–3^]	*d* [nm]	RMS [nm]	water CA [°]
**Film 3**	–327 ± 4	5.78 ± 0.06	1.0^21^	58 ± 0.6	4.22	95.1 ± 1.1
**Film 2**	–195 ± 6	3.46 ± 0.10	1.5^21^	23 ± 0.7	2.70	42.8 ± 1.2
**Film 2DHP**	–72 ± 11				41.2	51.0 ± 1.4

a RMS roughness was determined from AFM images. Water CA was obtained by goniometry.

A small increase in dissipation Δ*D* became visible for thicker films and its desilylated form (Figure 3, hollow gray triangle at 25 mg mL^–1^). On the one hand, increasing film thickness causes a more viscoelastic behavior compared to a purely elastic thin layer. On the other hand, more hydrophilic cellulose ferulate films obtained by desilylation are containing adsorbed water. Those films are inherently softer than TMS cellulose ferulate films. However, in practice, films can be considered as rigid if Δ*F*/Δ*D* > 25.^24^ The Sauerbrey relationship is valid for the whole data set of Figure 3.

**Figure 4:**
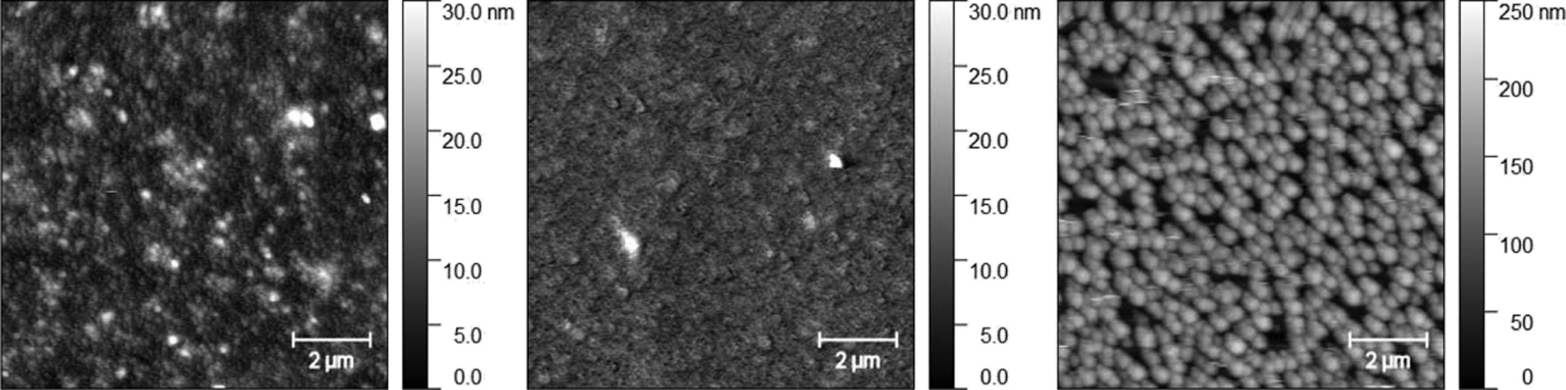
AFM images (10 μm × 10 μm) of **Film 3** (left), **Film 2** (center), and **Film 2DHP** (right) recorded in the contact mode.

AFM measurements show smooth surfaces before and after desilylation (Table 1, Figure 4). The surface morphology of **Film 2** and **3** is very similar. A few nanometric particles are visible on both surfaces. **Film 2** appears with softer edges in the topography due to a condensed layer structure after cleavage of TMS groups. However, RMS roughness did not change significantly from 4.2 nm to 2.7 nm.

According to the expectations, water CA is high (95°) for TMS cellulose ferulate films, possessing hydrophobic silyl groups and also hydrophobic ferulate moieties. After cleavage of TMS groups, the water CA decreases to 43°. Compared to the water CA of a pure cellulose film (25–30°), the value for **Film 2** is higher due to remaining hydrophobic ferulate groups.

**Figure 5:**
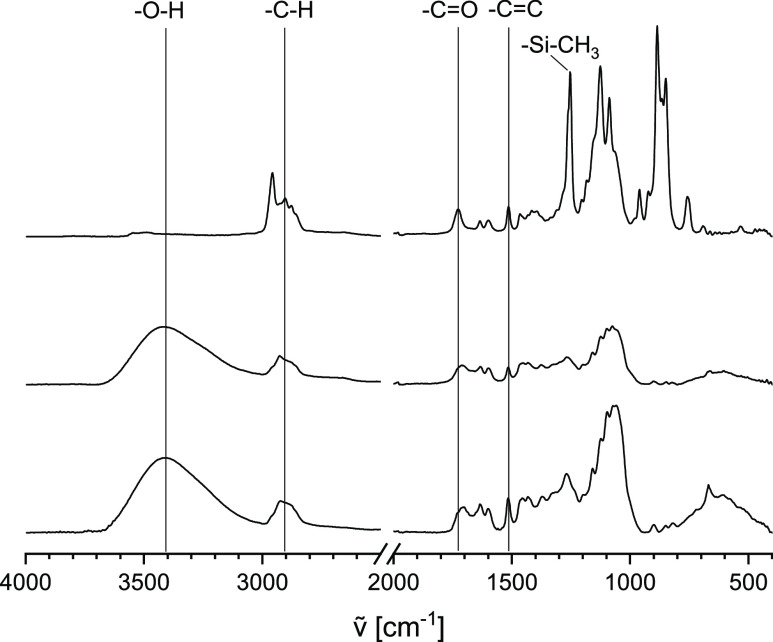
FTIR spectra of **Film 3** (top), **Film 2** (center), and **Film 2DHP** (bottom).

The FTIR spectrum of **Film 3** (TMS cellulose ferulate, Figure 5) shows intense aliphatic −C–H vibrations from TMS groups. Moreover, the typical peak of the Si–CH_3_ vibration at 1250 cm^–1^ is visible. The ferulate moieties are indicated by the −C=O signal at 1722 cm^–1^. In addition, the resonance at 1510 cm^–1^ shows aromatic structures. After desilylation (**Film 2**), OH stretching appears due to the formation of aliphatic and aromatic hydroxyl groups. The intensity of aliphatic −C–H vibrations is decreased, and in particular, Si–CH_3_ vibration at 1250 cm^–1^ disappears. Signals arising from ferulate groups are still visible.

### Artificial Lignification of Thin Films

Oxidative polymerization of coniferyl alcohol on cellulose ferulate films was carried out in aqueous H_2_O_2_ solutions catalyzed by HRP adsorbed on the surface. To underline the reactivity of anchor groups (ferulate) on the films, experiments were run in parallel with pure cellulose layers, obtained by regeneration of TMS cellulose films. The effect of the covalent attachment of artificial lignin to the surface via lignin-carbohydrate complex, that is ferulic acid ester, could be visualized on silicon wafers.

**Figure 6:**
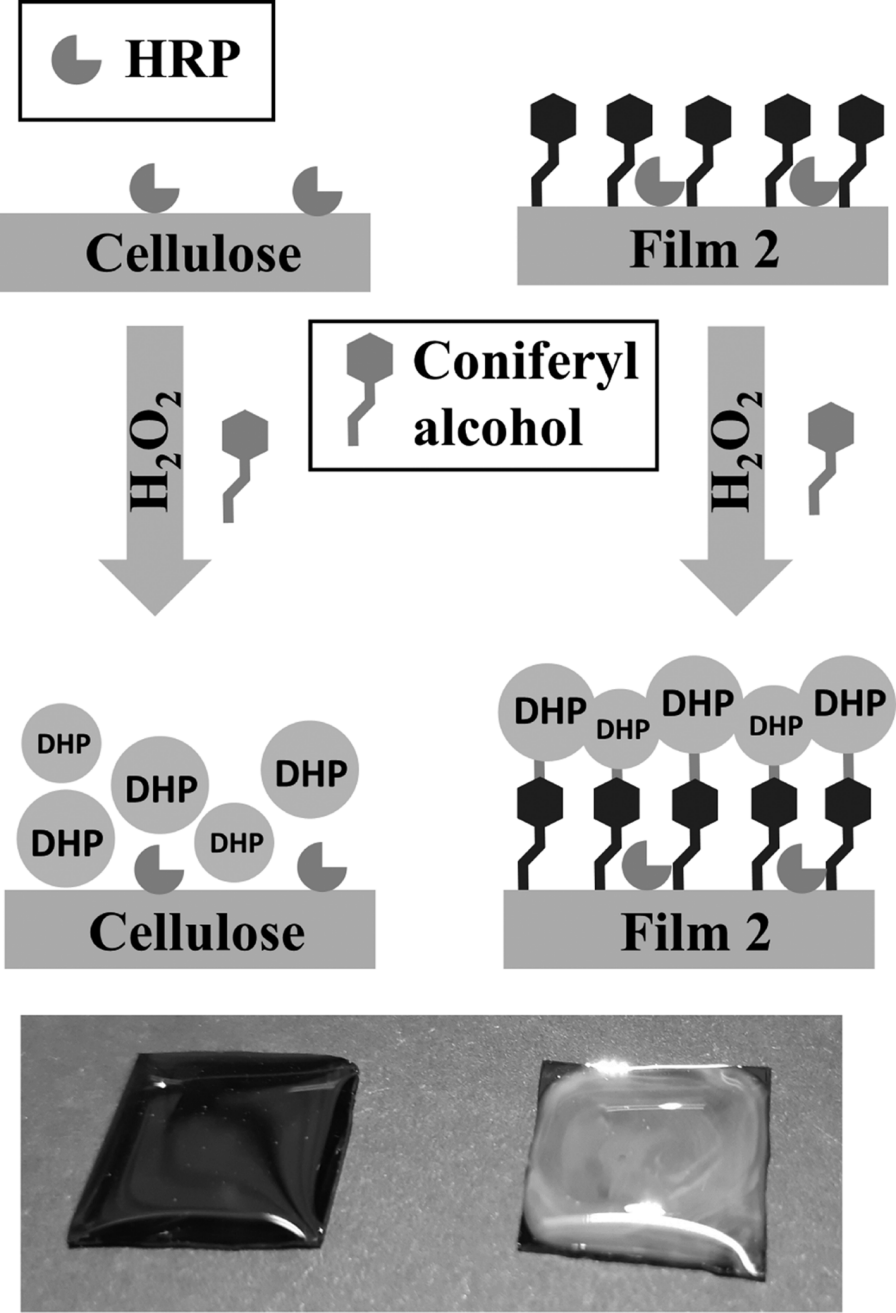
Schematic illustration of the lignification process on pure cellulose films (left) and cellulose ferulate films (right).

Figure 6 shows a schematic illustration of the lignification process on pure cellulose films (left) and cellulose ferulate films (right) including the photographical images of wafers (15 mm × 15 mm). In the first step, wafers were pretreated with HRP solution and subsequently rinsed with water to remove loosely bound enzyme. Afterward, aqueous coniferyl alcohol/H_2_O_2_ solutions were placed on the films to perform polymerization. It could be already seen by naked eye that a bright layer was formed on the cellulose ferulate film. Images from light microscopy (1000-fold magnification, Figure S4) show a uniform topography composed of nanometric globules. The supramolecular structures are also stable after rinsing with water and blow drying. In contrast to cellulose ferulate films, DHP are not tightly bound to pure cellulose films. The wafer appears to be optically similar to the bare cellulose film after rinsing.

**Figure 7:**
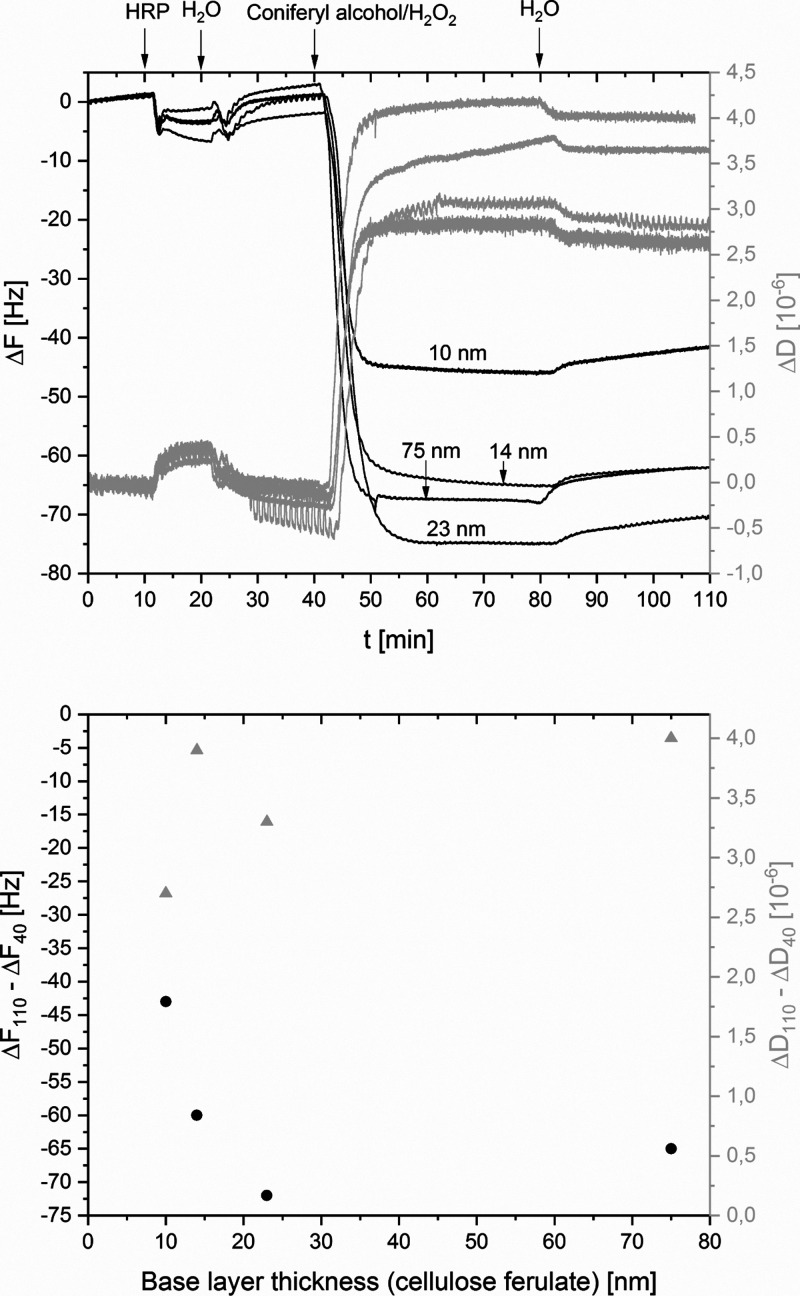
Top: QCM-D flow-through experiments (Δ*F*—black, Δ*D*—gray) of the polymerization of coniferyl alcohol. Inlet of reagents and curves arising from different base layer thicknesses are marked. Base layer thickness of cellulose ferulate films in nanometer is individually labeled. Bottom: Δ*F*_110_ – Δ*F*_40_ (black) and Δ*D*_110_ – Δ*D*_40_ (gray) of QCM-D flow-through experiments evaluated at 40 min and 110 min, respectively, show dependency of polymerization on base layer thickness of cellulose ferulate.

For advanced real-time measurements, polymerization of coniferyl alcohol on thin films was carried out on QCM-D sensors in a flow-through experiment. Figure 7 (top) shows Δ*F* and Δ*D* values over time. The inlet of HRP, coniferyl alcohol/H_2_O_2_, and rinsing steps are marked. The concentrations of reagents applied for artificial lignification in a QCM-D instrument were already optimized by Wang et al.^16^ However, the focus of our study is to discover novel films for lignin polymerization. Therefore, we used concentrations from their previous work,^16^ that is 1 mg mL^–1^ HRP, 0.5 mg mL^–1^ coniferyl alcohol, and 20 mM H_2_O_2_. The adsorption of HRP on the surface could be detected but not quantified by QCM-D. Δ*F* is too small during adsorption and rinsing, which is compensated by inherent baseline drifts. However, enzyme activity per area was determined by ABTS assay and is 650 U m^–2^.

The introduction of coniferyl alcohol/H_2_O_2_ into the flow cell containing a cellulose ferulate-coated sensor with adsorbed HRP lead to a rapid change in frequency, indicating the increase in areal mass, that is the formation of DHP on the surface. For quantitative evaluation of the polymerization, the difference of Δ*F* and Δ*D* at 40 min and 110 min (rinsing H_2_O) was considered. The shifts are indicated as Δ*F*_110_ – Δ*F*_40_ and Δ*D*_110_ – Δ*D*_40_ in Figure 7 (bottom) but named as Δ*F*_DHP_ and Δ*D*_DHP_ hereafter. Thus, bulk effects from coniferyl alcohol and H_2_O_2_ could be removed; moreover, loosely attached DHP does not contribute to the areal mass. However, layers of DHP show pronounced irreversible deformations under oscillating stress, that is, Δ*D*_DHP_ increases up to 4 × 10^–6^, and the Sauerbrey equation is not valid (Δ*F*/Δ*D* < 25). A subsequent change from the inlet of coniferyl alcohol and H_2_O_2_ to rinsing with H_2_O has only minor influence on Δ*F* and Δ*D*. There is just a small amount of DHP that could be removed from the surface, and the remaining layer behaves soft. This is in accordance with the work of Wang et al.^16^ who also noticed that the Sauerbrey relationship is not valid for DHP films. Thus, within this study, we did not calculate areal mass densities but compared Δ*F*_DHP_ values. Nevertheless, the dependency of Δ*F*_DHP_ from layer thickness of cellulose ferulate films indicates an interesting polymerization behavior (Figure 7, bottom). For cellulose ferulate films, a thickness of 14 to 75 nm leads to equal Δ*F*_DHP_ values in the range of −60 to −70 Hz. Since the standard deviation of measurements is 11 Hz, the differences are not significant. Only for a very low-film thickness of 10 nm, lignification leads to a frequency shift of −43 Hz. Since polymerization of coniferyl alcohol is mostly independent of film thickness of cellulose ferulate, it can be assumed that lignification takes place only at ferulate groups of the top layer of the film and not inside the material. This hypothesis is also supported by AFM images (Figure 4, right), showing nanospheres of about 300 nm in diameter on the surface (**Film 2DHP**).

Polymerization on pure cellulose films (thickness 15 nm, Table S1) without ferulate groups was not reproducible, that is, Δ*F* was around −7 Hz or −80 Hz depending on random adhesion (Figure S3). Successful lignification experiments of Wang et al.^16^ on films of cellulose nanocrystals can be explained by residual amounts of hemicellulose and lignin from spruce softwood pulp, which most likely provide anchor groups for polymerization. Thus, our study is not in conflict with previous work.

Surface roughness (RMS) of thin films increased from 2.7 to 41 nm (**Film 2DHP**) during lignification due to the formation of a particulate topography. The size of nanospheres on the surface is in accordance with supramolecular structure of DHP. A general accepted mechanism for the formation of DHP consists of four steps. First, modules are formed by polymerization of about 20 phenylpropanoid units followed by building up macromolecules with about 500 units,^27^ which are also called supermodules.^28^ Applying advanced microscopic techniques, an aggregation of these supermodules into globules and finally self-assembling into a colloidal crystal structure was found.^29^ The first two steps are based on the formation of covalent bonds, whereas the formation of supramolecular structures (step 3 and 4) is caused by hydrogen bonding and van der Waals interactions.^30^

In our study, we assume a covalent attachment of modules or supermodules to ferulate anchor groups on top of the films. Thus, supramolecular architecture is tightly linked to the surface instead of pure physical adsorption of DHP nanoparticles. The uniform structure of the nanoparticle layer indicates that lignification started from single polymerization centers on the surface and not from deposition of a DHP suspension.^29^

The water CA was increased from 44 to 51°due to the attachment of hydrophobic DHP. However, as observed by AFM, the layer of nanospheres is not completely covering the cellulose ferulate film, and an influence of the base layer might affect hydrophilic–hydrophobic properties of the film.

### Molecular Structure of Dehydrogenation Polymers

Layers of DHP on cellulose ferulate films were characterized by FTIR spectroscopy (Figure 5, bottom). However, the difference of the spectrum of **Film 2DHP** compared to that of **Film 2** is not pronounced. Thus, the formation of additional aromatic structures besides ferulic acid ester of cellulose cannot be detected unambiguously.

**Figure 8:**
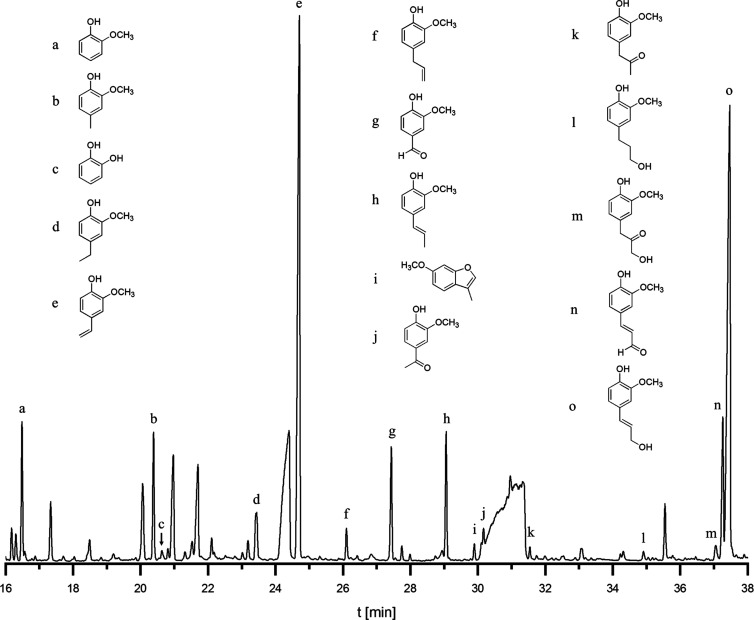
Chromatogram of pyrolysis products from **Film 2DHP** obtained by the Py-GC-MS measurement. Lignin-derived vaporable compounds (a–o) are assigned.

To investigate the molecular structure of artificial lignin in detail, **Film 2DHP** was pyrolyzed, and evaporable compounds were separated and identified with GC–MS, according to a procedure described for kraft lignin.^31^Figure 8 shows the chromatogram of a Py-GC-MS measurement and the assignment of lignin-derived compounds. The signals representing the fragments of cellulose are present but not labeled. A prominent peak with high intensity arises from coniferyl alcohol (**o**), which is the dominant product of the primary pyrolysis reaction (200–400 °C) of β-O-4 lignin models under H-donation.^32^ This is consistent with results for DHP obtained by Zutropfverfahren (ZT) where β-O-4 linkages are predominant.^33^ The products of Zulaufverfahren are rich in 5–5 structures. As the immobilized HRP is continuously supplied with the substrate, in this study, the reaction conditions can be considered as variation of the ZT procedure.^16^

The products of typical side chain conversion of coniferyl alcohol in primary pyrolysis reactions such as coniferyl aldehyde (**n**), dihydroconiferyl alcohol (**l**), isoeugenol (**h**), and 4-vinylguaiacol (**e**) could be found too.^34^ Moreover, other evaporable typical lignin pyrolysis products, for example, guaiacylacetone (**k**), acetovanillone (**j**), vanillin (**g**), eugenol (**f**), 4-ethylguaiacol (**d**), 4-methylguaiacol (**b**), and guaiacol (**a**) were detected.^35^ In addition to the β-O-4 motive, β-5 linkages were evidenced by pyrolysis product (**i**) arising from phenylcoumaran units. Catechol (**c**) is a product of secondary pyrolysis reactions (>400 °C), changing the aromatic substitution pattern.^32^

## Conclusions

TMS cellulose ferulate with DS values of 0.35 (ferulate) and 2.53 (TMS) was obtained by innovative Mitsunobu chemistry and subsequent conversion with HMDS. Silylation was required for spin coating of the cellulose derivative from easily evaporable solvents, for example, ethyl acetate. The obtained thin films could be desilylated by hydrochloric acid vapor to yield ferulate moieties on a cellulose surface. Thin films were characterized by FTIR spectroscopy, QCM-D, AFM, and goniometry. Layer thickness could be adjusted from 10 to 75 nm by varying mass concentrations of the polymer solution.

Dehydrogenative polymerization of coniferyl alcohol (0.5 mg mL^–1^) was carried out in the presence of H_2_O_2_ (20 mM) and catalyzed by HRP (650 U m^–2^) adsorbed on the surface. This procedure can be considered as variation of the ZT in a QCM-D device to allow online monitoring. The frequency shifts caused by formation of DHP on the sensors indicate an independency from base layer thickness in the range from 14 nm to 75 nm. Thus, lignification seems to take place only at ferulate groups of the top layer of the film but not inside the material. A referential experiment on a cellulose film without ferulate groups showed loose attachment of DHP on the surface. QCM-D measurements on pure cellulose layers were not reproducible since deposited material could be rinsed off.

AFM images show a supramolecular DHP layer composed of nanospheres with a diameter of about 300 nm on the cellulosic films. The molecular structure of DHP was investigated by Py-GC-MS to evidence β-O-4 and β-5 linkages. Based on a very uniform and adjustable molecular and supramolecular structure of the model films, biomimetic lignification experiments will be performed in future to discover structure–property relationships. Cellulose ferulate films will open new avenues to tailor advanced materials. Moreover, highly defined DHP layers could serve as sensors for ligninolytic enzymes.

## Supporting Information Available

FTIR spectra of **2** and **3**; NMR spectrum; referential QCM-D experiments; analysis of thin films; and image from light microscopy of DHP (PDF)
