# Supplementary material for: Growing of Artificial Lignin on Cellulose Ferulate Thin Films
Source: Biomacromolecules. 2022 Apr 19;23(5):2089–97. doi: 10.1021/acs.biomac.2c00096 (PMC9907350; doi:10.1021/acs.biomac.2c00096)
Supplement: Supplementary file 1 — bm2c00096_si_001.pdf [file bm2c00096_si_001.pdf]

Growing of artificial lignin on cellulose ferulate thin films

Thomas Elschner,[*,†] Jörg Adam,[‡] Hans Lesny,[‡] Yvonne Joseph,[‡] and Steffen Fischer[†]

† *Institute of Plant and Wood Chemistry, Technische Universität Dresden, Pienner Str. 19, 01737 Tharandt, Germany*

‡ *Institute of Electronic and Sensor Materials, TU Bergakademie Freiberg, Gustav-Zeuner-Str. 3, 09599 Freiberg, Germany*

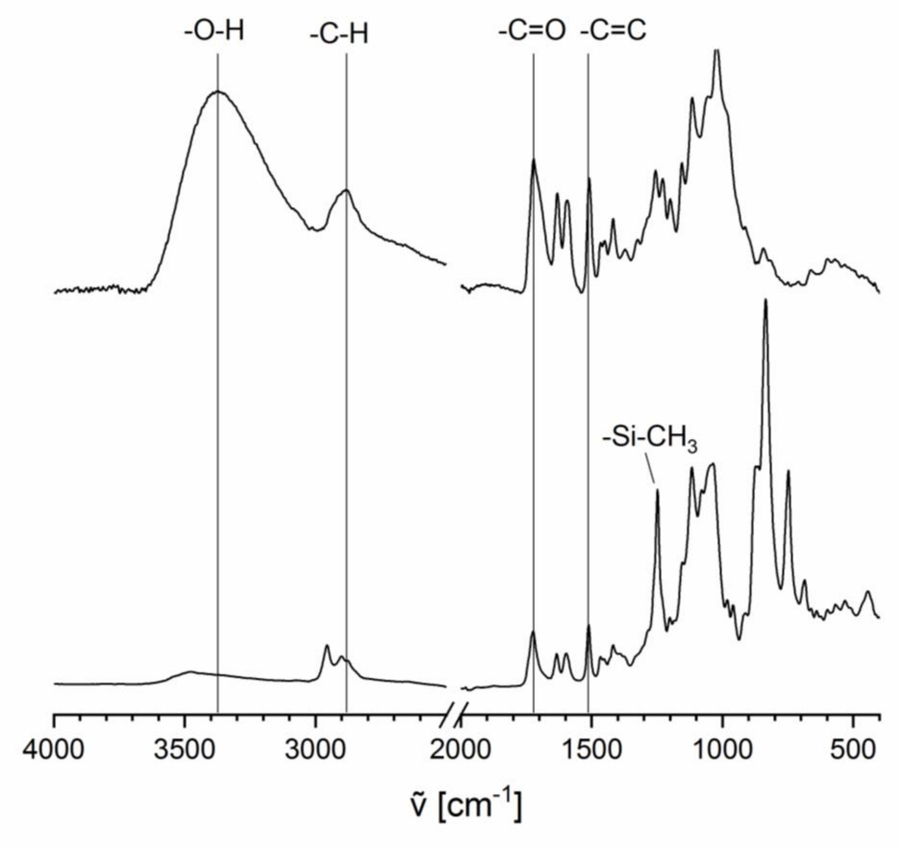

Figure S1: FTIR spectra of cellulose ferulate (**2**, top) and trimethylsilyl (TMS) cellulose ferulate (**3**, bottom).

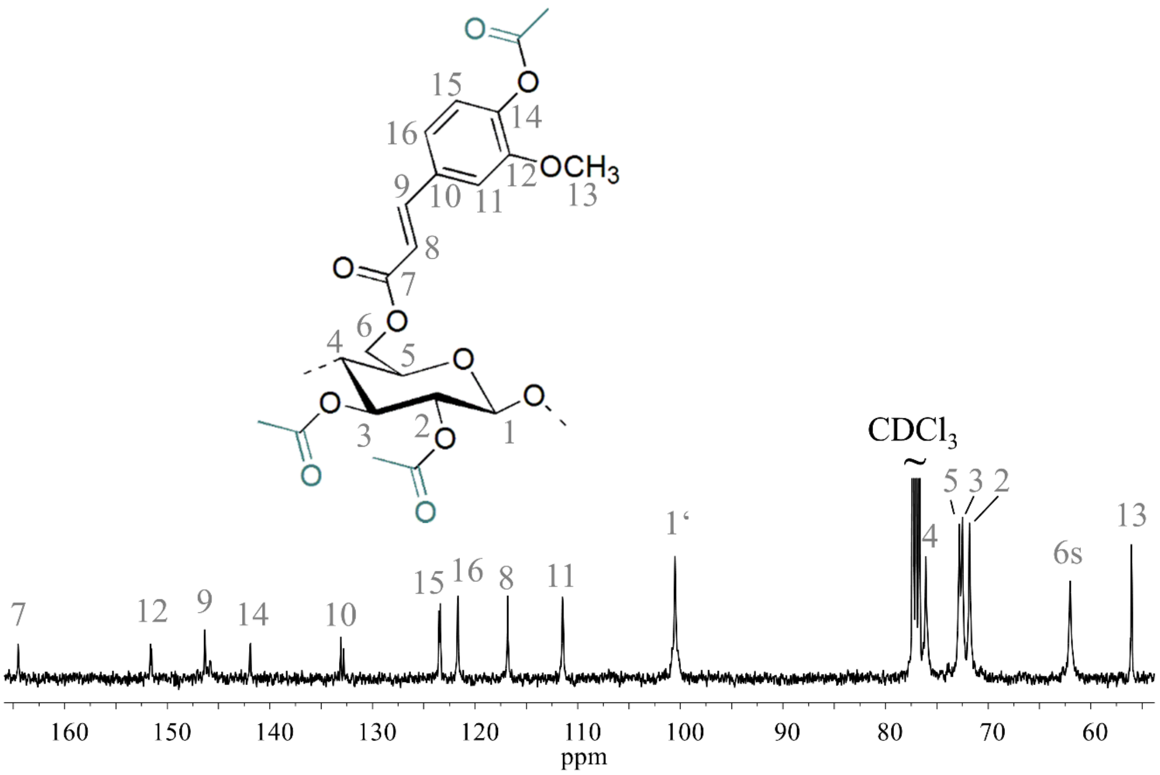

Figure S2: Section of the ^{13}C NMR spectrum of peracetylated cellulose ferulate recorded in $CDCl_3$ (adapted [26]).

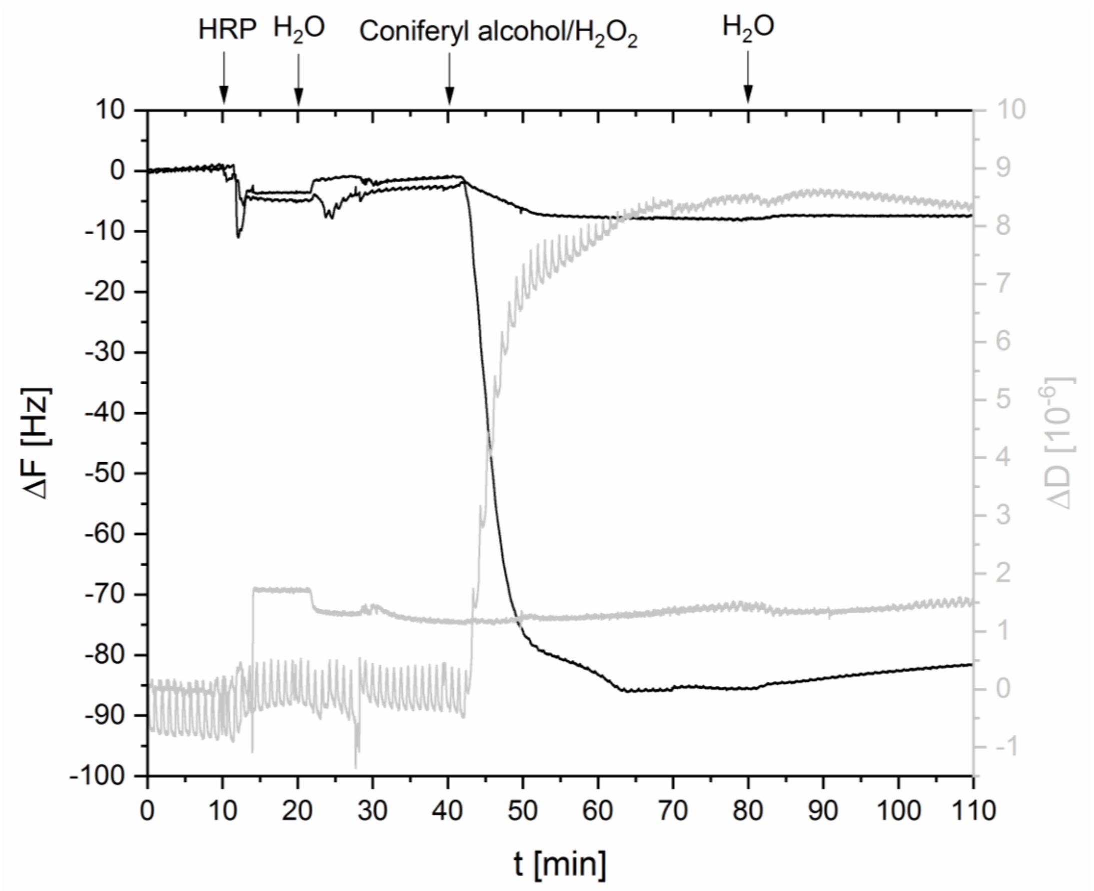

Figure S3: QCM-D experiments on pure cellulose films (ΔF –black; ΔD –gray): deposition of dehydrogenation polymers is not reproducible.

Table S1: Analysis of thin films of trimethylsilyl (TMS) cellulose and cellulose.

Sample	ΔF [Hz]	m_{film} [μgcm^{-2}]	ρ_{film} [gcm^{-3}]	d [nm]	Water CA[°]
TMS cellulose film	-217	3.85	1.0	38	93
Cellulose film	-126	2.23	1.5	15	31

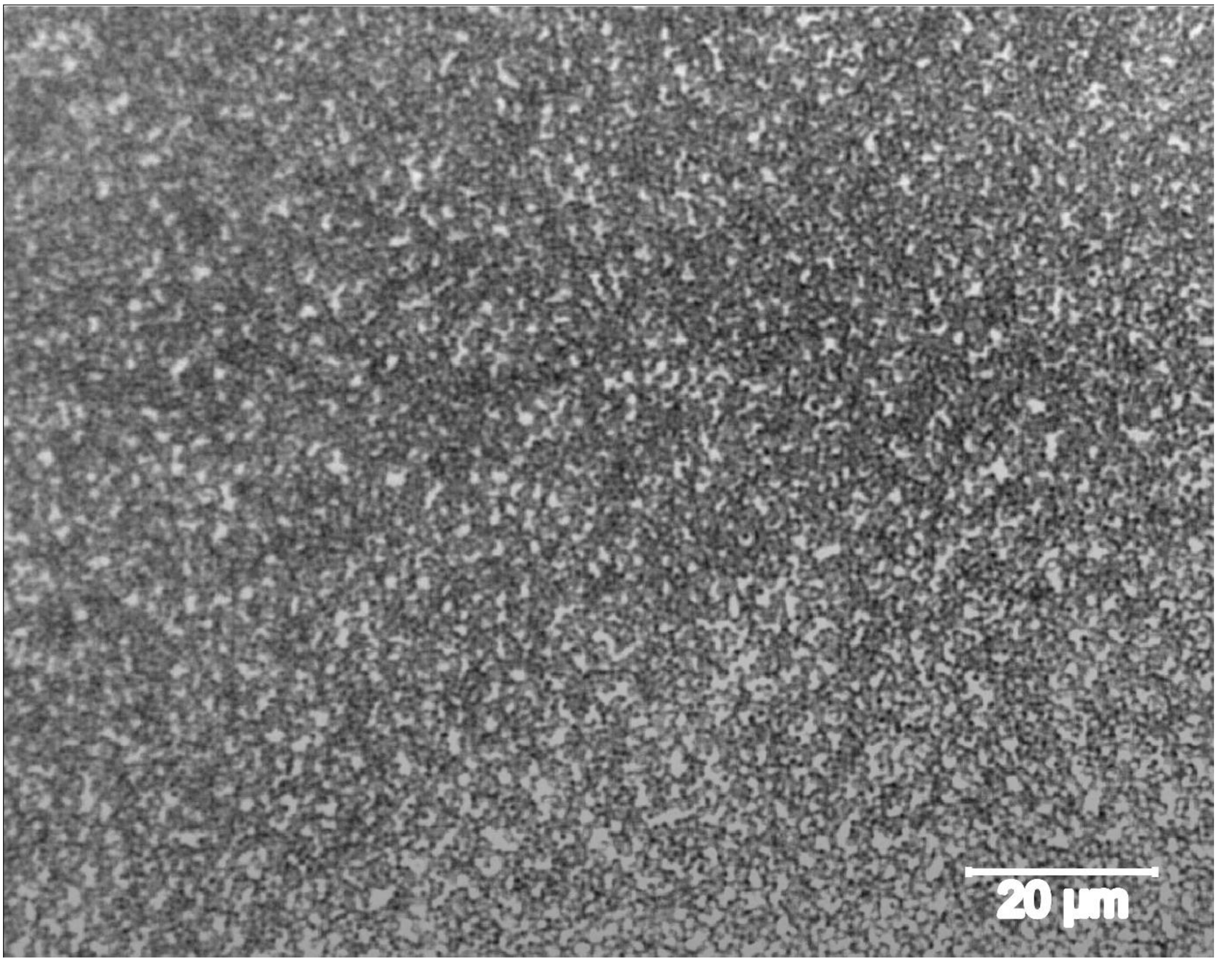

Figure S4: Image from light microscopy (1000-fold magnification) of **Film 2DHP** on silcon wafer.
